# Efficient implementation of the linear layer of block ciphers with large MDS matrices based on a new lookup table technique

**DOI:** 10.1371/journal.pone.0304873

**Published:** 2024-06-21

**Authors:** Tran Thi Luong, Nguyen Van Long, Bay Vo

**Affiliations:** 1 Academy of Cryptography Techniques, Hanoi, Vietnam; 2 Institute of Cryptographic Science and Technology, Hanoi, Vietnam; 3 Faculty of Information Technology, HUTECH University, Ho Chi Minh City, Vietnam; Jaramogi Oginga Odinga University of Science and Technology, KENYA

## Abstract

Block cipher is a cryptographic field that is now widely applied in various domains. Besides its security, deployment issues, implementation costs, and flexibility across different platforms are also crucial in practice. From an efficiency perspective, the linear layer is often the slowest transformation and requires significant implementation costs in block ciphers. Many current works employ lookup table techniques for linear layers, but they are quite costly and do not save memory storage space for the lookup tables. In this paper, we propose a novel lookup table technique to reduce memory storage when executing software. This technique is applied to the linear layer of block ciphers with recursive Maximum Distance Separable (MDS) matrices, Hadamard MDS matrices, and circulant MDS matrices of considerable sizes (e.g. sizes of 16, 32, 64, and so on). The proposed lookup table technique leverages the recursive property of linear matrices and the similarity in elements of Hadamard or circulant MDS matrices, allowing the construction of a lookup table for a submatrix instead of the entire linear matrix. The proposed lookup table technique enables the execution of the diffusion layer with unchanged computational complexity (number of XOR operations and memory accesses) compared to conventional lookup table implementations but allows a substantial reduction in memory storage for the pre-computed tables, potentially reducing the storage needed by 4 or 8 times or more. The memory storage will be reduced even more as the size of the MDS matrix increases. For instance, analysis shows that when the matrix size is 64, the memory storage ratio with the proposed lookup table technique decreases by 87.5% compared to the conventional lookup table technique. This method also allows for more flexible software implementations of large-sized linear layers across different environments.

## 1. Introduction

A block cipher is a cryptosystem that encrypts information using fixed-length data blocks. Shanon [1] proposed two design principles for block ciphers [2–4]: diffusion and confusion. Confusion involves forming a connection between the key and plaintext that is as intricate as possible, while diffusion is the second attribute that addresses the statistical correlation between the ciphertext and the plaintext, ensuring that the repetition in the plaintext is dispersed and eliminated throughout every bit of the ciphertext. In practice, to achieve these two properties block ciphers often use sboxes [5–8], with high nonlinearity for the nonlinear layer and MDS matrices [9–12] for the linear layer. In addition to block ciphers, there are also several other interesting research directions at the moment, such as ensuring data privacy and security in various environments [13–17], and secure signature and authentication schemes based on lattices in a quantum environment [18–21].

In a block cipher, the sboxes are often chosen to be small in size to reduce implementation costs, and the linear layer is utilized simultaneously with the sboxes to spread out the nonlinearity they provide. From an efficiency perspective, the linear layer is usually the slowest transformation and requires a lot of implementation costs in block ciphers. As a result, investigating the choice of the linear layer is not only directly linked to the ability of block ciphers to resist attacks, including those involving differential and linear attacks, but also affects the performance of the ciphers. At the same time, the study of the linear layer implementation method is becoming increasingly necessary for the performance of block ciphers.

In applied cryptography, block ciphers must not only ensure security but must also be flexible in implementation on different platforms. At present, the implementation is often applied to modern block ciphers when combining both linear and nonlinear layers at the same time as in AES [22, 23], GOST R 34.12–2015 [24], LED [25], and so on. It becomes apparent that as the size of the linear layer increases to guarantee adequate security, the computational complexity, and especially *the memory complexity* increases significantly. This problem may affect flexibility when implementing ciphers in environments with different computing power and resources.

In recent years, and mainly to gain advantages in hardware implementation, there have been many studies focusing on building recursive linear layers [26–29]. Accordingly, their linear matrix is a power of a companion matrix [30, 31] that has a simple form and is easy for hardware implementation. In [32], the authors proposed and presented a series of methods using the recursive property for linear matrices in the Kuznyechik cipher proposed by researchers from the FSB Agency in the Russian Federation. The method of using the byte-oriented lookup table technique of AES is also considered. The implementation executes on a platform with 64-bit registers and a memory size of 128 KB.

There have been a large number of studies focused on generating small-size recursive MDS matrices, such as MDS matrices with a size of 4. These matrices have advantages in hardware implementation [31, 33, 34]. However, software implementation exploiting the recursiveness of these matrices does not seem to have received much attention, especially in terms of using pre-computed lookup tables.

Another research direction is building linear layers using MDS matrices with the particular form of circulant matrices [35–38] or Hadamard matrices [39–41]. Although these matrices are not recursive, they have the feature that the number of elements in each column and each row in the matrix is the same. This property is fully exploited for hardware implementation, while in software implementation the lookup table technique mainly applies to small-size matrices, for example, 4×4 or 8×8. However, as the size of the linear matrix increases, there will be problems related to the memory needing to store the pre-computed lookup tables. This issue seems to be a limitation when implementing large matrices in environments with limited computing power.

### 1.1. Our contributions

In this article we exploit the properties of linear layers with the recursive MDS matrices, Hadamard MDS matrices, or the circulant MDS matrices, investigate some of the properties of the matrices and then propose a new lookup table technique to reduce the storage memory when executing in the software.

- Accordingly, the proposed lookup table technique exploits the recursive property of linear matrices, the similarity in elements in each row of the Hadamard matrix or circulant MDS matrix, thereby allowing the construction of a lookup table not for the entire linear matrix but only for a submatrix of it, while still yielding outputs with unchanged computational complexity and significantly reduced memory storage. The proposed method allows the implementation of a diffusion layer with the same computational complexity (number of XORs and memory accesses) as compared to the usual lookup table implementation. It allows us to significantly reduce the memory needed to store the pre-computed lookup tables, which can halve, or even decrease by 4, 8, or more times the memory needed when storing pre-calculated data compared to the methods of creating a lookup table for the entire MDS matrix in the linear layer, as in [23, 32, 42]. The larger the size of the MDS matrices of the linear layer, the more advantages the proposed method has, and thus the less storage memory it needs.
- It is quite possible to apply the proposed approach to the case of a matrix for a complete block to increase the diffusion layer’s branch number, and in such cases, the security of the block cipher is increased without increasing the memory needed for the pre-calculated lookup tables.
- The proposal also allows for more flexible software implementation of a large linear layer in different environments. These results are significant in practice when implementing the linear layer of block ciphers using a large MDS matrix, and high security for block ciphers can be ensured when using these matrices.
- This technique is applied to the linear layer of block ciphers and hash functions with MDS matrices of considerable sizes.

The structure of the rest of the article is as follows. In Section 2, related works are presented. Section 3 introduces the linear layer using recursive MDS matrices, Hadamard MDS matrices, and circulant MDS matrices. Section 4 shows how to use the lookup table for linear layers with representation through a linear matrix over a finite field. Section 5 proposes an efficient method to execute a large-size linear layer with recursive MDS, Hadamard MDS, and circulant MDS matrices based on a lookup table. Section 6 presents some analysis and comparisons, while Section 7 is the conclusion of this work.

## 2. Related works

The main focus of this section is to present studies on the implementation issues and block ciphers’ linear layer.

At present, researchers are particularly interested in recursive MDS matrices [26–29, 33] due to their sparse form as powers of a companion matrix [30, 31]. Therefore, it is very convenient to implement this kind of matrix in both hardware and software. In [26], the authors presented a direct building method for recursive MDS matrices based on a specific recursive structure. The authors provided examples of constructed matrices and evaluated their performance in terms of encryption and decryption. They compared their method with other existing methods and demonstrated its effectiveness in terms of computational complexity and storage requirements. In [27], the authors provided conditions for diagonal and non-singular companion matrices to become recursive MDS matrices. The authors then proposed methods to build recursive MDS matrices using Gabidulin codes and Bose–Chaudhuri–Hocquenghem (BCH) codes. In [28], the authors studied how to implement encryption and decryption in similar Linear Feedback Shift Register (LFSR) circuits for the diffusion stage of a block cipher. The authors proposed a specific form of recursive MDS matrix to perform nearly identical encryption and decryption and analyze some experimental results. In [29], the authors presented a very sparse and diagonal type of matrix. From there, they gave the minimum fixed number of XORs of the MDS matrices generated from the above matrix form. The authors also proposed recursive matrices of sizes 4, 5, 6, and 7 that are very lightweight, along with their corresponding number of XORs. In [33], the authors introduced a novel diffusion layers class that is highly effective, consisting of multiple Feistel-like structures. They investigated some linear functions to reach the optimal branch number for the diffusion layer of 4×4 words. Additionally, they expanded their results for 8×8 word diffusion layers. However, the documents [26–31, 33] only focus on the construction of efficient recursive MDS matrices and do not discuss how to implement or efficiently execute these matrices. Specifically, they do not explore software implementations based on lookup tables for these matrices.

Another research direction focuses on circulant MDS matrices [35–38]. In [35], the authors presented a novel algebraic proof that demonstrates the infeasibility of involutory circulant MDS matrices with coefficients in fields of characteristic 2. For odd characteristics, the authors established parameters that may enable the existence of such matrices. They introduced a novel method for directly building θ-circulant MDS matrices that are almost involutory and showed that these have potential applications in hardware executions. In [36], the authors focused on building effective circulant MDS matrices of size *k*, where *k* is less than or equal to 8, and investigating their inverses, which are crucial for SPN block ciphers. The authors also examined the practical and interesting attributes of the matrices. They proved that involutory circulant MDS matrices are impossible, and demonstrated that circulant matrices of size 2^*k*^×2^*k*^ cannot be both MDS and orthogonal. In [37], the authors presented and proposed some effective circulant MDS matrices of size *d*, with *d* values up to 8, and their inverses. They investigated some intriguing and valuable properties of circulant matrices. They also showed that involutory circulant MDS matrices do not exist and that an orthogonal and MDS 2^*d*^×2^*d*^ circulant matrix is impossible. In [38], the authors presented a method for computing the inverses of 2^*n*^×2^*n*^ (*n*≥3) circulant matrices with entries in *GF*(2^*m*^). Their approach involved utilizing a program to create the cofactors of an 8×8 circulant matrix, which was used to recursively build the inverse of any circulant one.

Hadamard matrices [39–41] are also a special form of MDS matrix and are of current research interest. In [39], the authors addressed presented methods of building lightweight MDS matrices, specifically focusing on 4×4 involutory MDS matrices that can be realized with the smallest XOR number over *GF*(2^8^). The authors gave some techniques to obtain involutory MDS matrices of size 4 with few XOR operations. In [40], the authors of this earlier research paper presented a novel method for creating Hadamard matrices, known as the Ghadamard matrix (the generalized Hadamard), and applied it to the construction of MDS matrices that are involutory and lightweight. They extended their method to any matrix of size *k*×*k*, with any *k*, and obtained some involutory MDS matrices of sizes 4 and 8 over *GF*(2^4^) and *GF*(2^8^). In [41], the authors introduced two novel methods for constructing MDS matrices. Firstly, they suggested a method for creating involutory MDS matrices using Vandermonde matrices. Secondly, they presented a new way for building Hadamard MDS matrices of size 2^*n*^×2^*n*^ over *GF*(2^*m*^). As we know, the implementation method using lookup tables is completely independent of the matrix form. Therefore, the studies [35–41] choose matrices with special forms (Circulant, Hadamard, or recursive) to optimize hardware implementations or bit-slice implementations in software for resource-constrained environments. However, these documents also do not explore the aspect of software implementation based on lookup tables for these matrices or methods for saving memory storage.

In [22, 23], the authors presented the AES algorithm and the implementation technique for this algorithm, including the technique of using pre-computed tables. These lookup tables combine both nonlinear (S-box) and linear transformations. The results of their approach make the AES algorithm highly implementable in software, on 32-bit platforms. This method of executing AES lookup tables was later exploited and applied by many algorithms, such as the Streebog hash function [42], Kalyna block cipher [43, 44], Kuznyechik block cipher [24, 32, 42], GOST R 34.11–2012 hash function [45], Whirlpool hash function [46, 47], and so on. This combination uses a pre-calculated lookup table. AES uses a linear layer with a 4×4 MDS matrix, and the required lookup table size is 8 KB [23], and for GOST R 34.11–2012 [45] the necessary lookup table size is 128 KB for a matrix of size 16×16. For the above two ciphers, with a 128-bit block size, their number of memory accesses is 16, while the number of XORs of 32-bit numbers (for AES) or 64-bit numbers (for Kuznyechik) are 16 and 32, respectively.

The implementation method using the lookup technique is also considered, but the approach to compute the lookup tables is based entirely on the AES implementation principle described in [23]. In [32], the authors presented some approaches to implementing the recursive linear layer based on the companion matrices. Specifically, they analyzed the implementation for the linear layer of the Kuznyechik block cipher, in which the software execution method exploits the characteristics of the linear feedback shift registers. In [42], the authors presented the method of using the pre-calculated lookup table to implement and evaluate the performance of the entire Kuznyechik algorithm. The implementation is executed and compiled on a 64-bit platform.

In [23, 32, 42], the authors all used the technique of lookup tables, but no one has yet exploited the specific attributes of the companion matrix, the Hadamard matrix, and the circulant matrix to reduce stored memory during execution methods. However, the aforementioned settings only apply to small linear layers of 128-bit size.

The common characteristic of the works [22–25, 32, 42–44, 46, 47] is that they implement the lookup table method for software using the full linear matrix (referred to as the conventional lookup table technique) of these ciphers, thus not saving memory storage. Moreover, these implementations only apply to small linear layers with sizes less than or equal to 128 bits. Table 1 shows the lookup table size, corresponding memory space, and related parameters for the linear matrix when applying the full matrix lookup table implementation in these documents.

**Table 1:** Implementation parameters based on lookup tables for linear matrices in [22–25, 32, 42–44, 46, 47].

№	Cipher	Matrix size, Finite field	Register size (bits)	Lookup table size	Number of lookup tables	Number of memory accesses	Number of XORs	Size of the linear transform
1	LED block cipher [25]	4×4, *GF*(2^4^)	16	128 B	4	4	4	16 bits
2	AES block cipher [22, 23])	4×4, *GF*(2^8^)	32	4 KB	4	4	4	32 bits
3	Whirlpool [46, 47], Streebog [42], Kalyna [43, 44])	8×8, *GF*(2^8^)	64	16 KB	8	8	8	64 bits
4	Kuznyechik [24, 32]	16×16, *GF*(2^8^)	64	64 KB	32	32	32	128 bits

Hereby, it is evident that the lookup table implementations in these documents do not save memory storage for the lookup tables, especially when the size of the linear matrix is large. We will address this issue in this paper and provide a comparison table of our results with the implementation parameters in [22–25, 32, 42–44, 46, 47] at the end of the paper.

In this paper, we investigate some characteristics of the recursive MDS linear layers, the linear layers using the Hadamard MDS matrices or the circulant MDS matrices, and then propose an efficient software implementation method based on a lookup table for large linear layers using MDS matrices of these types.

## 3. The linear layer of block ciphers uses the Hadamard matrix, the recursive matrix, and the circulant matrix

In this section, we introduce some commonly used MDS matrices for the linear layer of block ciphers, such as the Hadamard MDS matrix, the recursive MDS matrix, and the circulant MDS matrix. We assume that MDS matrices are used for the linear layer of block ciphers, for the sake of simplicity.

### 3.1. Recursive linear layer based on companion matrix

In the context of this paper, we consider the linear layer obtained from a linear matrix *M* of size *m* over a finite field *GF*(2^*n*^), where *m* is even. Its recursiveness is expressed in the fact that the matrix *M* is determined by the *m* power of a *m*×*m* companion matrix *A* of the following form:

$$A=\left(\begin{array}{ccccc} 0 & 0 & \cdots & 0 & a_{0}\\ 1 & 0 & \cdots & 0 & a_{1}\\ 0 & 1 & \cdots & 0 & a_{2}\\ \vdots & \vdots & \ddots & \vdots & \vdots \\ 0 & 0 & \cdots & 1 & a_{m-1} \end{array}\right),a_{i}\in GF(2^{n}),0\leq i\leq m-1$$ (1)

The linear layer in this paper is denoted as *L*: *V*_*mn*_→*V*_*mn*_, specified as the right multiplication of row vector *X* by matrix *M* and the output is row vector *Y*, as follows:

$$Y=X\times M=X\times A^{m}=(((\cdots ((X\times \underset{m}{\underset{︸}{A)\times A)\times \ldots \times A)\times A)\times A}}),$$ (2)

where $Y=(y_{0},y_{1},\ldots ,y_{m-1}),X=(x_{0},x_{1},\ldots ,x_{m-1}),x_{i},y_{i}\in GF(2^{n})$.

### 3.2. Linear layer using a Hadamard matrix

The advantage of implementing Hadamard matrices is that they can be used to design the linear layer of cryptographic primitives. In this section, we will briefly introduce this matrix form.

The Hadamard matrix of size *m* is a matrix of the following form:

$$H=\left(\begin{array}{ll} H_{1} & H_{2}\\ H_{2} & H_{1} \end{array}\right)$$ (3)

Where the matrices *H*_1_ and *H*_2_ are square matrices of size $\frac{m}{2}$.

Consider this example with the matrices: $H_{1}=\left(\begin{array}{ll} a_{0} & a_{1}\\ a_{1} & a_{0} \end{array}\right),H_{2}=\left(\begin{array}{ll} a_{2} & a_{3}\\ a_{3} & a_{2} \end{array}\right),$ there is a 4×4 Hadamard matrix as follows:

$$H=\left(\begin{array}{llll} a_{0} & a_{1} & a_{2} & a_{3}\\ a_{1} & a_{0} & a_{3} & a_{2}\\ a_{2} & a_{3} & a_{0} & a_{1}\\ a_{3} & a_{2} & a_{1} & a_{0} \end{array}\right)$$ (4)

### 3.3. Linear layer using circulant matrix

A circulant matrix is one obtained by rotating one element in each row or column. For example, a circulant matrix of size 4×4 has the following form:

$$C=\left(\begin{array}{llll} a_{0} & a_{1} & a_{2} & a_{3}\\ a_{3} & a_{0} & a_{1} & a_{2}\\ a_{2} & a_{3} & a_{0} & a_{1}\\ a_{1} & a_{2} & a_{3} & a_{0} \end{array}\right)$$ (5)

#### Remark

See that for two kinds of Hadamard or circulant matrices, the set of elements in each of their rows or columns is the same. We can use this property for hardware as well as software implementations for linear layers using these matrix forms.

## 4. Linear layer implementation using the lookup table technique

In this section, we show how to implement a linear layer using the lookup table technique. Denote the linear layer by a linear transformation *L* representing a right-hand multiplication of a row vector *X* by a matrix *M* over *GF*(2^*n*^). Then the linear transformation *L*: *V*_*mn*_→*V*_*mn*_ can be expressed as follows:

$$L(X)=X\times M=(x_{0},x_{1},\ldots ,x_{m-1})\times \left(\begin{array}{cccc} b_{0,0} & b_{0,1} & \cdots & b_{0,m-1}\\ b_{1,0} & b_{1,1} & \cdots & b_{1,m-1}\\ \vdots & \vdots & \ddots & \vdots \\ b_{m-1,0} & b_{m-1,1} & \cdots & b_{m-1,m-1} \end{array}\right)$$ (6)

where $M={\left(b_{i,j}\right)}_{m\times m},b_{i,j}\in GF(2^{n}),i,j=0,1,\ldots ,m-1$, and $X=(x_{0},x_{1},\ldots ,x_{m-1})\in V_{mn}$. Consider the linear transformation $L_{i}^{*}:\;V_{n}\to V_{mn},i=\bar{0,m-1}$:

$$\forall x\in V_{n}:L_{i}^{*}=x\cdot b_{i,0}∥x\cdot b_{i,1}∥\ldots ∥x\cdot b_{i,m-1}.$$ (7)

where the operation ∥ is the concatenation.

From (6) and (7), for all $X=(x_{0},x_{1},\ldots ,x_{m-1})\in V_{mn}$, we have:

$$\begin{array}{c} Y=L(X)=L_{0}^{*}(x_{0})⊕L_{1}^{*}(x_{1})⊕\ldots ⊕L_{m-1}^{*}(x_{m-1})=\\ =|\begin{array}{c} x_{0}\cdot b_{0,0}∥x_{0}\cdot b_{0,1}∥\ldots ∥x_{0}\cdot b_{0,m-1}\\ ⊕\\ x_{1}\cdot b_{1,0}∥x_{1}\cdot b_{1,1}∥\ldots ∥x_{1}\cdot b_{1,m-1}\\ ⊕\\ \ldots \\ ⊕\\ x_{m-1}\cdot b_{m-1,0}∥x_{m-1}\cdot b_{m-1,1}∥\ldots ∥x_{m-1}\cdot b_{m-1,m-1} \end{array}| \end{array}$$ (8)

Thus, the transformation *L*: *V*_*mn*_→*V*_*mn*_ can be represented through *m* linear transformations *L*_0_,…,*L*_*m*−1_, each *L*_*i*_ can be calculated through a lookup table of 2^*n*^ rows, each row of *mn* bit size, and these lookup tables are arranged in ascending order as follows:

$$\left|\begin{array}{ccccccc} b_{i,0}\cdot (0) & ∥ & b_{i,1}\cdot (0) & ∥ & \cdots & ∥ & b_{i,m-1}\cdot (0)\\ b_{i,0}\cdot (1) & ∥ & b_{i,1}\cdot (1) & ∥ & \cdots & ∥ & b_{i,m-1}\cdot (1)\\ & \cdots & & & \cdots & & \\ b_{i,0}\cdot (r) & ∥ & b_{i,1}\cdot (r) & ∥ & \cdots & ∥ & b_{i,m-1}\cdot (r)\\ & \cdots & & & \cdots & & \\ b_{i,0}\cdot (2^{n}-1) & ∥ & b_{i,14}\cdot (2^{n}-1) & ∥ & \cdots & ∥ & b_{i,0}\cdot (2^{n}-1) \end{array}\right|$$ (9)

where the multiplication is performed in *GF*(2^*n*^).

Thus the *r*-th row of this table is determined by:

$$L_{i}^{*}(r)=b_{i,0}\cdot (r)∥b_{i,1}\cdot (r)∥\ldots ∥b_{i,m-1}\cdot (r),\forall r\in GF(2^{n}),i=0,1,\ldots ,m-1.$$

The same process is performed for the linear transformation *L*^−1^: *V*_*mn*_→*V*_*mn*_, but in this case using matrix *M*^−1^. We do not describe this process in detail here.

Thus, implementing *L*: *V*_*mn*_→*V*_*mn*_ requires *m* memory accesses storing *m* lookup tables and *m* XORs of *mn*-bit words.

A lookup table like this can also be applied to concurrently combine two nonlinear and linear transformations for SPN ciphers, as in AES [22, 23], GOST R 34.12–2015 [24], and so on. Accordingly, we can create the number of lookup tables here as *m* tables, with the total required memory size of *m*^2^×*n*×2^*n*^ bits. Details of the settings for each value of *m*, *n* are in [23, 32, 42].

For example, for *m* = 32, *n* = 8 (applicable to SPN ciphers with the block size of *m*×*n* = 256 bits), the required memory is 32^2^×8×2^8^ bits = 256 KB. If applied to the decryption process, the memory needed is double this. In this example, considering the computing power of common computers, each lookup table cannot be stored by 256-bit numbers. Therefore, instead of having to set up 32 lookup tables, we need to set up 32×4 = 128 lookup tables whose elements in each table are 64-bit numbers (64-bit is the length of registers of common computers today). With such an increase in the number of lookup tables, and depending on the actual computing power, the number of memory accesses will increase, and the number of XORs of 64-bit numbers will increase significantly. This increase affects the execution speed, and this is an optimization problem in the implementation of large linear layers. In the next section, we propose some approaches to the specific form of the MDS matrix *M* used in the linear layer reducing the number of lookup tables.

## 5. A novel approach for implementing large linear layers efficiently utilizing the lookup table technique

This section describes a novel approach for implementing a large linear layer using the lookup table technique where a linear layer can use the recursive MDS matrices, the circulant MDS matrices, or the Hadamard MDS matrix. The proposed method can halve, or even reduce by 4, 8, or even more times, the memory needed when storing pre-calculated data compared to methods making a lookup table for the entire matrix, as mentioned in [23, 32, 42].

### 5.1. For the recursive linear layer

For linear layers using recursive MDS matrices (2), instead of making a lookup table for the matrix *M* = *A*^*m*^, we propose an implementation method by creating a lookup table for the matrix *A*^*m*/2^. To make it easier and more intuitive, we consider the case of the small linear layers as *m* = 4 and *n* = 4. Then consider the companion matrix of the following form:

$$A=\left(\begin{array}{llll} 0 & 0 & 0 & a_{0}\\ 1 & 0 & 0 & a_{1}\\ 0 & 1 & 0 & a_{2}\\ 0 & 0 & 1 & a_{3} \end{array}\right),\mathrm{for}\;a_{i}\in GF(2^{4}),0\leq i\leq 3.$$ (10)

Then,

$$A^{2}=\left(\begin{array}{cccc} 0 & 0 & a_{0} & a_{0}a_{3}\\ 0 & 0 & a_{1} & a_{0}⊕a_{1}a_{3}\\ 1 & 0 & a_{2} & a_{1}⊕a_{2}a_{3}\\ 0 & 1 & a_{3} & a_{2}⊕a_{3}^{2} \end{array}\right),M=A^{4}=\left(\begin{array}{cccc} a_{0} & a_{0}a_{3} & * & *\\ a_{1} & a_{0}⊕a_{1}a_{3} & * & *\\ a_{2} & a_{1}⊕a_{2}a_{3} & * & *\\ a_{3} & a_{2}⊕a_{3}^{2} & * & * \end{array}\right),$$ (11)

Here * is a certain value of *GF*(2^4^). We are only interested in the similarity of the last two columns and the first two columns in the two matrices *A*^2^ and *A*^4^, respectively. With such a representation, consider multiplying the right vector *X* = (*x*_0_, *x*_1_, *x*_2_, *x*_3_) by matrix *A*^2^, producing:

$$Y^{*}=(y_{0}^{*},y_{1}^{*},y_{2}^{*},y_{3}^{*})=X\times A^{2}=(x_{0},x_{1},x_{2},x_{3})\times \left(\begin{array}{cccc} 0 & 0 & a_{0} & a_{0}a_{3}\\ 0 & 0 & a_{1} & a_{0}⊕a_{1}a_{3}\\ 1 & 0 & a_{2} & a_{1}⊕a_{2}a_{3}\\ 0 & 1 & a_{3} & a_{2}⊕a_{3}^{2} \end{array}\right)$$

$$\Rightarrow \left\{ \begin{array}{l} y_{0}^{*}=x_{2}\\ y_{1}^{*}=x_{3}\\ y_{2}^{*}=a_{0}x_{0}⊕a_{1}x_{1}⊕a_{2}x_{2}⊕a_{3}x_{3}\\ y_{3}^{*}=a_{0}a_{3}x_{0}⊕(a_{0}⊕a_{1}a_{3})x_{1}⊕(a_{1}⊕a_{2}a_{3})x_{2}⊕(a_{2}⊕a_{3}^{2})x_{3} \end{array}\right.$$ (12)

and for matrix *A*^4^, producing:

$$Y=(y_{0},y_{1},y_{2},y_{3})=X\times A^{4}=(x_{0},x_{1},x_{2},x_{3})\times \left(\begin{array}{cccc} a_{0} & a_{0}a_{3} & * & *\\ a_{1} & a_{0}⊕a_{1}a_{3} & * & *\\ a_{2} & a_{1}⊕a_{2}a_{3} & * & *\\ a_{3} & a_{2}⊕a_{3}^{2} & * & * \end{array}\right)$$

$$\Rightarrow \left\{ \begin{array}{l} y_{0}=a_{0}x_{0}⊕a_{1}x_{1}⊕a_{2}x_{2}⊕a_{3}x_{3}\\ y_{1}=a_{0}a_{3}x_{0}⊕(a_{0}⊕a_{1}a_{3})x_{1}⊕(a_{1}⊕a_{2}a_{3})x_{2}⊕(a_{2}⊕a_{3}^{2})x_{3}\\ y_{2}=*\\ y_{3}=* \end{array}\right.$$ (13)

From (12) and (13), $y_{0}=y_{2}^{*}$ and $y_{1}=y_{3}^{*}$. The output of the recursive linear layer can thus use the matrix *A*^2^ to calculate the first half of the coordinate value of the output vector *Y* through the Y* vector, then apply the matrix *A*^2^ with the input vector of coordinates $\left(x_{2},x_{3},y_{2}^{*},y_{3}^{*}\right)$ to get half the value of the remaining coordinates of the *Y* vector.

In the general case, with the matrix *A* of size *m* over *GF*(2^*n*^), the way to set up the lookup table and use the lookup table to calculate the output vectors is described as follows. There are a total of *m* lookup tables, denoted by *T*_0_, *T*_1_,…*T*_*m*−1_, and each table has 2^*n*^ elements. Each element of the tables has a size equal to the concatenation size (the operation ∥) of half the number of entries on a row of the matrix equal to (*mn*)/2 bits.

Rewrite expression (2) as follows:

$$Y=(y_{0},y_{1},\ldots ,y_{m-1})=X\times A^{m}=\left((x_{0},x_{1},\ldots ,x_{m-1})\times A^{m/2}\right)\times A^{m/2}$$ (14)

From matrix *A* of the form (1), calculate matrix *A*^*m*/2^. Assuming it has the following type:

$$A^{m/2}=\left(\begin{array}{cccccc} 0 & \cdots & b_{0,j} & b_{0,j+1} & \cdots & b_{0,m-1}\\ 0 & \cdots & b_{1,j} & b_{1,j+1} & \cdots & b_{1,m-1}\\ \vdots & \ddots & \vdots & \vdots & \ddots & \vdots \\ 0 & \cdots & b_{m-1,j} & b_{m-1,j+1} & \cdots & b_{m-1,m-1} \end{array}\right),$$ (15)

where $b_{i,j}\in GF(2^{n}),i=0,1,\ldots ,m-1;\;j=\frac{m}{2},\frac{m}{2}+1,\ldots ,m-1$.

Consider the linear transformation: $L_{i}^{*}:\;V_{n}\to V_{\frac{mn}{2}},i=\bar{0,m-1}$:

$$\forall x\in V_{n}:L_{i}^{*}=x\cdot b_{i,j}∥x\cdot b_{i,j+1}∥\ldots ∥x\cdot b_{i,m-1},j=m/2.$$ (16)

From the above arguments, it can be seen that when multiplying vector *X* by matrix *A*^2^ we will get the coordinates in the first half of vector *Y*. From Formulas (14), (15), and (16) we can calculate the first half of the vector *Y* as follows:

$$\left(\begin{array}{c} y_{0}∥y_{1}∥\ldots ∥y_{\frac{m}{2}-1} \end{array})=L_{0}^{*}(x_{0})⊕L_{1}^{*}(x_{1})⊕\ldots ⊕L_{m-1}^{*}(x_{m-1}\right)$$

$$=\left|\begin{array}{c} x_{0}\cdot b_{0,j}∥x_{0}\cdot b_{0,j+1}∥\ldots ∥x_{0}\cdot b_{0,m-1}\\ ⊕\\ x_{1}\cdot b_{1,j}∥x_{1}\cdot b_{1,j+1}∥\ldots ∥x_{1}\cdot b_{1,m-1}\\ ⊕\\ \ldots \\ ⊕\\ x_{m-1}\cdot b_{m-1,j}∥x_{m-1}\cdot b_{m-1,j+1}∥\ldots ∥x_{m-1}\cdot b_{m-1,m-1} \end{array}\right|,j=\frac{m}{2}$$ (17)

Thus, the coordinate values $\left(y_{0},y_{1},\ldots ,y_{\frac{m}{2}-1}\right)$ can be calculated through expression (17) by *m* linear transformations (16). We can calculate each of these linear transformations through a table of 2^*n*^ elements. The size of each element of the table is equal to the concatenation size (operation ||) of half the number of entries on a row of the matrix and equal to (*mn*)/2 bits. These lookup tables are computed and sorted in ascending order of the value *x*.

The *i*-th lookup table is calculated as follows:

$$\left|\begin{array}{l} \;b_{i,j}\cdot 0\;∥b_{i,j+1}\cdot 0\;∥\ldots ∥b_{i,m-1}\cdot 0\\ \;\ldots \\ \;b_{i,j}\cdot k∥b_{i,j+1}\cdot k\;∥\ldots ∥b_{i,m-1}\cdot k\\ \;\ldots \\ b_{i,j}\cdot (2^{n}-1)∥b_{i,j+1}\cdot (2^{n}-1)\;∥\cdots ∥b_{i,m-1}\cdot (2^{n}-1) \end{array}\right|$$ (18)

Similarly, to calculate the coordinates in the second half of the *Y* vector, we just need to repeat the above process when multiplying the X* vector by the matrix *A*^2^, and the coordinates of this vector are:

$$X^{*}=(x_{\frac{m}{2}},x_{\frac{m}{2}+1},\ldots ,x_{m-1},y_{0},y_{1},\ldots ,y_{\frac{m}{2}-1})$$ (19)

The coordinates of the vector X* are the addresses of each lookup table (18).

The above analysis shows that the proposed method of using a lookup table to compute the value of the recursive linear layer *allows halving the memory when storing pre-calculated data* compared to creating a lookup table for the entire matrix as in [23, 32, 42]. The number of XORs in (17) is just adding modulo 2 of (*mn*)/2 numbers.

**Note:** The evaluation of the number of lookup tables here is only theoretical because, in practice for large linear layers, this number will change depending on the storage capacity (register size) of the implementation environment and the storage capacity of the compiler. We will analyze this in detail in Table 1.

For the recursive linear layer of a form (2), its inverse matrix is also of the same type, so we can use the above approach to calculate the *L*^−1^ transformation. We do not detail this issue here.

### 5.2. For linear layers using a circulant matrix

As analyzed in Section 2, circulant matrices have the same set of elements in each of their rows or columns. We use this feature to propose an implementation method using the lookup table technique. Just like in Section 4.1, for simplicity here we consider the circulant matrix of size 4 over *GF*(2^4^) as follows:

$$C=\left(\begin{array}{llll} a_{0} & a_{1} & a_{2} & a_{3}\\ a_{3} & a_{0} & a_{1} & a_{2}\\ a_{2} & a_{3} & a_{0} & a_{1}\\ a_{1} & a_{2} & a_{3} & a_{0} \end{array}\right)$$ (20)

A linear transformation: *L*: *V*_16_→*V*_16_.


$$Y=(y_{0},y_{1},y_{2},y_{3})=X\times C=(x_{0},x_{1},x_{2},x_{3})\times \left(\begin{array}{llll} a_{0} & a_{1} & a_{2} & a_{3}\\ a_{3} & a_{0} & a_{1} & a_{2}\\ a_{2} & a_{3} & a_{0} & a_{1}\\ a_{1} & a_{2} & a_{3} & a_{0} \end{array}\right)$$ (21)

From here we have:

$$y_{0}∥y_{1}=\left(\begin{array}{c} a_{0}x_{0}∥a_{1}x_{0}\\ ⊕\\ a_{3}x_{1}∥a_{0}x_{1}\\ ⊕\\ a_{2}x_{2}∥a_{3}x_{2}\\ ⊕\\ a_{1}x_{3}∥a_{2}x_{3} \end{array}\right),y_{2}∥y_{3}=\left(\begin{array}{c} a_{2}x_{0}∥a_{3}x_{0}\\ ⊕\\ a_{1}x_{1}∥a_{2}x_{1}\\ ⊕\\ a_{0}x_{2}∥a_{1}x_{2}\\ ⊕\\ a_{3}x_{3}∥a_{0}x_{3} \end{array}\right)$$ (22)

We see that, in (22), the formula for the sum of XORs of *y*_0_‖*y*_1_ and *y*_2_‖*y*_3_ have the same terms for the coefficients of the matrix *C*, which differ only in order. Therefore, if we use the lookup table method for half of the columns of matrix *C*, we can calculate all the output coordinates of the *Y* vector. Indeed, make 4 lookup tables for the concatenation of the last two columns of the matrix *C* as follows:

$$\begin{array}{c} T_{0}=\left|\begin{array}{ccc} a_{2}\cdot 0 & ∥ & a_{3}\cdot 0\\ \ldots & & \ldots \\ a_{2}\cdot k & ∥ & a_{3}\cdot k\\ \cdots & & \ldots \\ a_{2}\cdot (2^{4}-1) & ∥ & a_{3}\cdot (2^{4}-1) \end{array}\right|,T_{1}=\left|\begin{array}{ccc} a_{1}\cdot 0 & ∥ & a_{2}\cdot 0\\ \ldots & & \ldots \\ a_{1}\cdot k & ∥ & a_{2}\cdot k\\ \cdots & & \ldots \\ a_{1}\cdot (2^{4}-1) & ∥ & a_{2}\cdot (2^{4}-1) \end{array}\right|\\ T_{2}=\left|\begin{array}{ccc} a_{0}\cdot 0 & ∥ & a_{1}\cdot 0\\ \ldots & & \ldots \\ a_{0}\cdot k & ∥ & a_{1}\cdot k\\ \cdots & & \ldots \\ a_{0}\cdot (2^{4}-1) & ∥ & a_{1}\cdot (2^{4}-1) \end{array}\right|,T_{3}=\left|\begin{array}{ccc} a_{3}\cdot 0 & ∥ & a_{0}\cdot 0\\ \ldots & & \ldots \\ a_{3}\cdot k & ∥ & a_{0}\cdot k\\ \cdots & & \ldots \\ a_{3}\cdot (2^{4}-1) & ∥ & a_{0}\cdot (2^{4}-1) \end{array}\right| \end{array}$$ (23)

where 0≤*k*≤2^4^−1 and the multiplication is performed over *GF*(2^4^). Then *y*_0_‖*y*_1_ and *y*_2_‖*y*_3_ are determined by:

$$\begin{array}{c} y_{0}∥y_{1}=T_{2}[x_{0}]⊕T_{3}[x_{1}]⊕T_{0}[x_{2}]⊕T_{1}[x_{3}]\\ y_{2}∥y_{3}=T_{0}[x_{0}]⊕T_{1}[x_{1}]⊕T_{2}[x_{2}]⊕T_{3}[x_{3}] \end{array}$$ (24)

Therefore, in this case, we only have to set up 4 lookup tables, but the size of each lookup table is reduced by half compared to the way to set up the lookup table for the entire matrix as in Section 3. As a result, it can reduce the required memory by half. This result is similar to that of the recursive linear layer in Section 4.1.

In the general case, with a matrix *A* of size *m* over *GF*(2^*n*^), the way to create a lookup table and how to use it to compute the output vectors is generalized. This method requires all *m* lookup tables, denoted by *T*_0_, *T*_1_,…,*T*_*m*−1_. Each lookup table has 2^*n*^ elements, and each element of the table has a size equal to the concatenation size (operating ||) of half the number of entries per row of the matrix and it is (*mn*)/2 bits.

### 5.3. For linear layers using Hadamard matrix

The Hadamard matrix of size *m* is a matrix of the following form:

$$H=\left(\begin{array}{ll} H_{1} & H_{2}\\ H_{2} & H_{1} \end{array}\right)$$

We see that the set of elements in each row or column of this matrix is the same. The approach is thus the same as for the circulant matrix and can be applied to create lookup tables for half of the columns of *H*. The computational complexity, as well as the storage memory complexity, are also reduced similar to that seen with the linear layer using a circulant matrix.

## 6. Analysis and comparison

In this section, we analyze and compare the complexity of pre-calculated lookup tables for some specific cases of the recursive linear layers and linear layers based on the Hadamard matrix (denoted *H* matrix) or circulant matrix (denoted *C* matrix). Store each element in each lookup table in numbers up to 64 bits (this is the register size of a common computer).

For an *m*×*m* linear matrix *M* with elements over *GF*(2^*n*^), where *n* = 8 and *m* = 2^*l*^, *l*≥4 (here only matrices of large sizes are considered as the proposed method is only efficient for large sizes). Then if the register size of the underlying platform is *h* bits (*h* = 8, 16, 32, 64), a comparison table of general implementation parameters when using the proposed lookup table method and conventional lookup table is presented in Table 2.

**Table 2:** Comparison of general implementation parameters using the proposed lookup table method and conventional lookup table method.

Parameters	Method for implementation using the lookup table	Register size (bits)	Memory size requirement (in bits) for the lookup table	Number of lookup tables	Number of memory accesses	Number of XORs	Size of the linear transform
*m* = 2^*l*^, *l*≥4, *n* = 8	For matrix *M* (using the conventional lookup table)	*h*	*nm*^2^2^*n*^	$\frac{nm^{2}}{h}$	$\frac{nm^{2}}{h}$	$\frac{nm^{2}}{h}$	*mn*
	**For the matrix $A^{\frac{h}{n}}$, or for $\frac{h}{n}$ the number of columns of *M* (when *M* is a Hadamard or Circulant matrix)** **(using the proposed lookup table)**	*h*	*hm*2^*n*^	*m*	$\frac{nm^{2}}{h}$	$\frac{nm^{2}}{h}$	

Table 3 describes the complexity of the new implementation technique using lookup tables compared with the conventional lookup table techniques used in [22–25, 32, 42–44, 46, 47] for matrices of size *m* (*m* = 4, 8,16, 32, 64) over *GF*(2^*n*^).

**Table 3:** Comparison of the parameters of the implementation using the proposed lookup tables and ones in [22–25, 32, 42–44, 46, 47].

№	Parameters	Method for implementation using the lookup table	Register size (bits)	Lookup table size	Number of lookup tables	Number of memory accesses	Number of XORs	Size of the linear transform
1	*m* = 4 *n* = 4	For matrix *M* (For example, the matrix of LED [25])	16	128 B	4	4	4	16 bits
		**For matrix *A*** ^**2**^ **, half the number of columns of *H* or *C***	**8**	**64 B**	**4**	**8**	**8**	
2	*m* = 4 *n* = 8	For matrix *M* (For example, the matrix of AES [22, 23])	32	4 KB	4	4	4	32 bits
		**For matrix *A*** ^**2**^ **, half the number of columns of *H* or *C***	**16**	**2 KB**	**4**	**8**	**8**	
3	*m* = 8 *n* = 4	For matrix *M*	32	4 KB	8	8	8	32 bits
		**For matrix *A*** ^**4**^ **, half the number of columns of *H* or *C***	**16**	**2 KB**	**8**	**16**	**16**	
4	*m* = 8 *n* = 8	For matrix *M* (For example, the matrix of Whirlpool [46, 47], Streebog [42], Kalyna [43, 44])	64	16 KB	8	8	8	64 bits
		**For matrix *A*** ^**4**^ **, half the number of columns of *H* or *C***	**32**	**8 KB**	**8**	**16**	**16**	
5	*m* = 16 *n* = 8	For matrix *M* (For example, the matrix of Kuznyechik [24, 32])	64	64 KB	32	32	32	128 bits
		**For matrix *A*** ^**8**^ **, half the number of columns of *H* or *C***	**64**	***32 KB***	***16***	**32**	**32**	
6	*m* = 32 *n* = 8	For matrix *M*	64	256 KB	128	128	128	256 bits
		**For matrix *A*** ^**8**^ **, a quarter of number of columns of *H* or *C***	**64**	***64 KB***	***32***	**128**	**128**	
7	*m* = 64 *n* = 8	For matrix *M*	64	1024 KB	512	512	512	512 bits
		**For matrix *A*** ^**8**^ **, one per eight of number of columns of *H* or *C***	**64**	***128 KB***	***64***	**512**	**512**	

In Table 3, we can see that when the linear layer size is less than 128 bits, the complexity of the implementation using the matrix *M* according to the usual lookup table technique and that of the proposed method is different. In this case, the memory required according to the proposed method is less than half that of the usual one, and the number of XORs of the proposed method is twice as many as the usual one when making a lookup table for the matrix *M*. Note the lines highlighted in black in Table 3 represent the results of the proposed method.

When the linear layer size is 128 bits, the proposed method only needs 16 lookup tables, half of the 32 tables of the usual one using a lookup table for the matrix *M*. For the proposed method, the lookup table size is still less than half that of the usual one. We can explain this difference as follows. For the usual implementation method using a lookup table for the matrix M, according to the analysis in Section 3, only 16 tables are needed, but the size of each element in the table is 128 bits. This value needs to be divided into two 64-bit numbers for today’s conventional computers. Therefore, instead of setting up 16 tables, 32 tables are required, depending on the register size of the actual software execution environment. However, the proposed method only creates a lookup table for half of the columns of matrix *A*^8^ or half of the columns of the matrix *H* or *C*. Thus only 16 lookup tables are needed for these cases.

Using Table 3, we can further analyze some cases to compare the proposed lookup table method and the conventional lookup table method.

✓ In row 2 (*m* = 4, *n* = 8) of Table 3, this is a case where the MixColumns matrix of AES is an example. In this case, the memory storage used for the proposed lookup table is 2 KB, whereas the conventional lookup table requires 4 KB. Therefore, the proposed method needs 50% less memory storage. However, in this case, the matrix size is small (*m* = 4), so the number of memory accesses and XOR operations of the proposed method is higher than with the conventional lookup table method.
✓ In row 4 (*m* = 8, *n* = 8) of Table 3, this is the case associated with the linear layer parameters of the Whirlpool hash function [46, 47], Streebog hash function [42], Kalyna block cipher [43, 44]). In this case, the memory storage used for the proposed lookup table is 8 KB, whereas the conventional lookup table requires 16 KB. The proposed method also saves 50% less memory storage. Similarly to the previous case, due to the small matrix size (*m* = 8), the number of memory accesses and XOR operations of the proposed method is higher than with the conventional lookup table method.
✓ In row 5 (*m* = 16, *n* = 8) of Table 3, this is the case where the linear layer parameters of Kuznyechik [24] serve as an example. If the register size is 64 bits as shown in Table 2, then the proposed method also uses 50% less memory storage (32 KB instead of 64 KB), whereas if implemented on a 32-bit register, the memory storage needed will decrease by 75%. In this case, however, the number of memory accesses and XOR operations of the proposed lookup table method are equivalent to those of the conventional lookup table method.
✓ In row 7 (*m* = 64, *n* = 8) of Table 3, when using a 64-bit register, the memory storage needed decreases by 87.5% (meaning only 128 KB is needed to store the proposed lookup table, instead of 1024 KB required for the conventional lookup table). Meanwhile, the number of memory accesses and XOR operations of the proposed lookup table method remains equivalent to those of the conventional lookup table method.

With these results, it can thus be observed that as the size of the linear layer matrix increases, the proposed lookup table technique becomes more advantageous, capable of significantly reducing the memory storage needed for the lookup table.

As the linear layer size increases, for example to 256 and 512 bits, we can use matrices that are not *A*^*m*/2^, but *A*^*m*/4^ = *A*^8^ and *A*^*m*/8^ = *A*^8^ respectively, for the recursive linear layer case. Similarly, for *H* or *C* matrices, we will create a lookup table for 1/4 of the columns or 1/8 of their columns. We do this so that the size of each element in each table matches the 64-bit register size. In this case, the memory size is reduced not only by 2 times but by 4 and 8 times. The number of tables created is also reduced in this example by 4 and 8 times, respectively.

Table 4 indicates the lookup table size (KB) with corresponding parameters. Table 5 shows a number of lookup tables with corresponding parameters.

**Table 4:** Lookup table size (KB) with corresponding parameters for *n* = 8, m = 2^*l*^.

	*l*	4	5	6	7
	Full Matrix	64	256	1024	4096
*h* = 32	one per eight of number of columns	16	32	64	128
*h* = 64	Half the number of columns	32	64	128	256

**Table 5:** Number of lookup tables with corresponding parameters for *n* = 8.

	*l*	4	5	6
	Full Matrix	32	128	512
*h* = 32	one per eight of number of columns	16	32	64
*h* = 64	Half the number of columns	16	32	64

We can understand flexibility here as being able to implement software implementation on platforms with different register sizes. We can choose the appropriate solution depending on the platform’s memory, storage resources, and register size.

**Fig 1:**
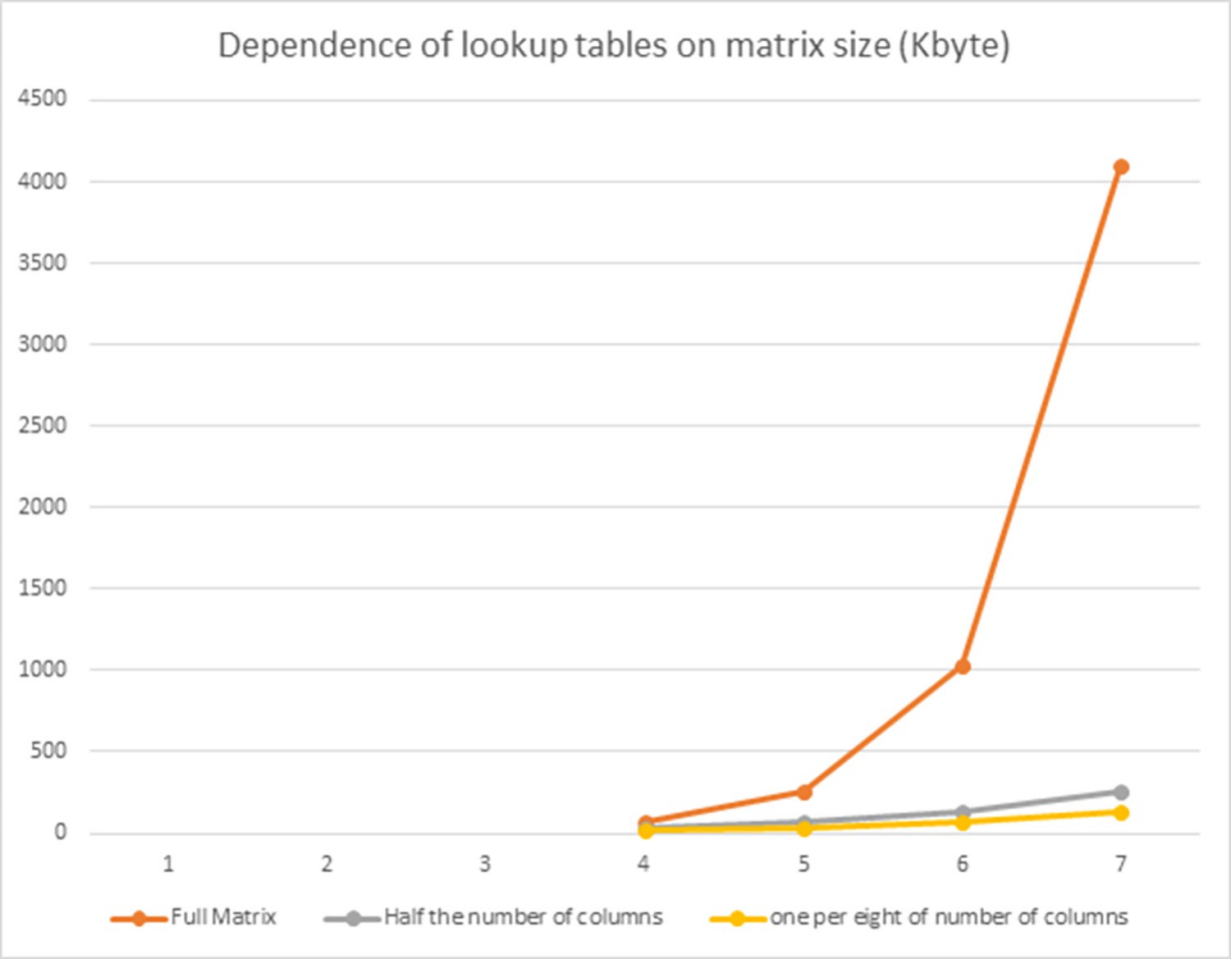
Correlation between matrix size and lookup table size (in Kbytes).

**Fig 2:**
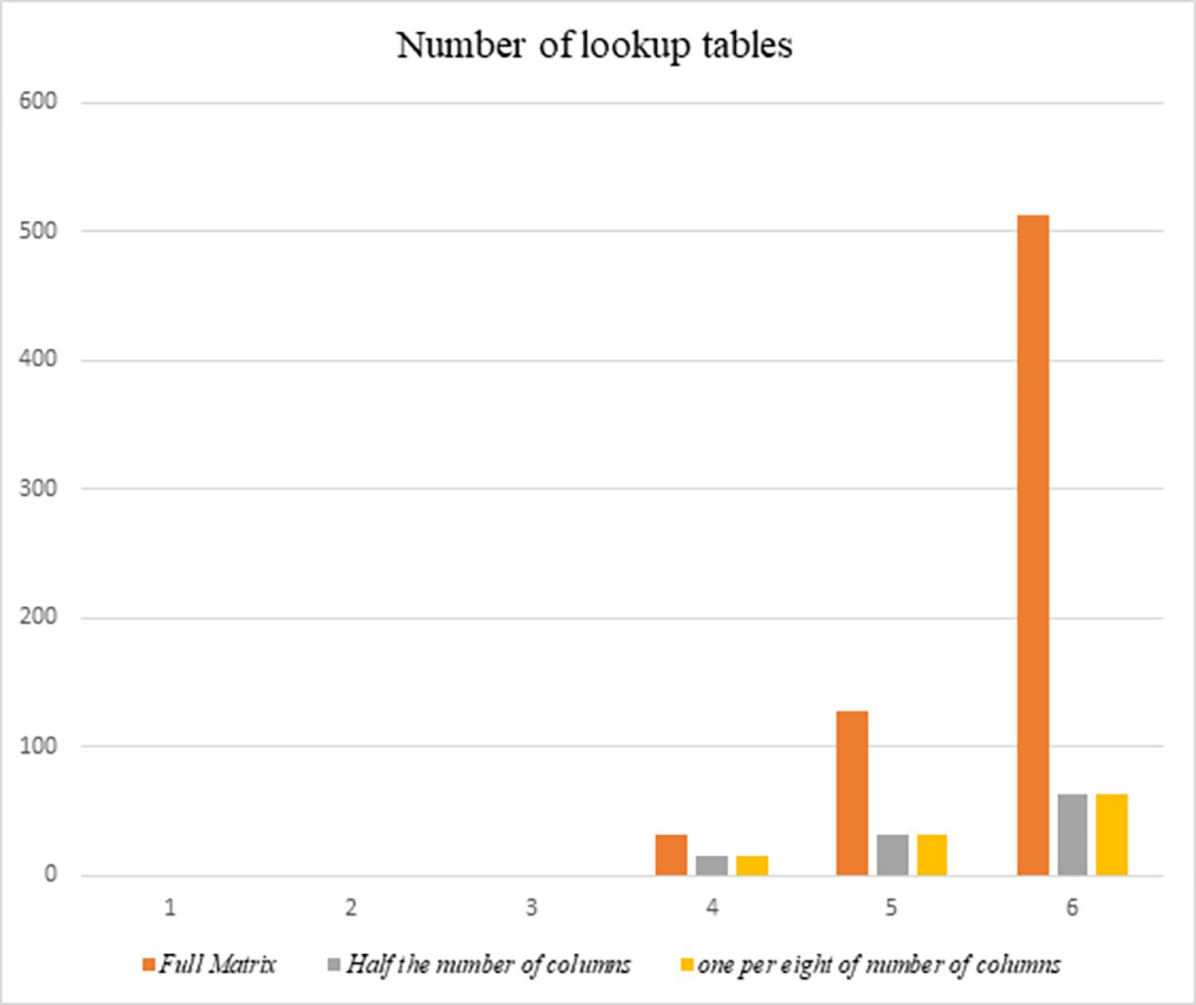
Number of lookup tables for various implementation types.

We illustrate with Fig 1 to depict between lookup table size and matrix size (in Kbytes) and Fig 2 to show the number of lookup tables for various implementation types.

Through Fig 1, we can observe that as the size of the linear matrix increases, with the conventional lookup table technique using the full matrix (red line), the size of the lookup table increases significantly and rapidly, resulting in a substantial increase in memory storage space for the lookup table. However, with our proposed lookup table technique (purple and beige lines), as the size of the linear matrix increases, the size of the lookup table increases insignificantly and remains small, indicating a minimal increase in memory storage. Therefore, applying our proposed lookup table technique is highly effective for large linear matrices, resulting in significant savings in memory storage space.

From Fig 2, it is evident that as the size of the linear matrix increases, the number of lookup tables increases substantially and rapidly with the conventional lookup table technique using the full matrix (red line), leading to significant consumption of memory storage space for these tables. Conversely, with our proposed lookup table technique (purple and beige lines), the increase in the number of lookup tables as the size of the linear matrix increases is minimal, indicating only a small increment in memory storage requirements. Hence, the application of our proposed lookup table technique proves highly effective for large linear matrices, resulting in considerable savings in memory storage space.

Below, we analyze some limitations of the proposed lookup table technique.

### 6.1. Limitations of the proposed method

As outlined in the original problem statement, the proposed approach is tailored for large-scale linear layers. While it can also be adapted to smaller linear layers, the analysis indicates its inefficacy in such cases. This can be viewed as a limitation of the proposed method. To illustrate this further, consider the example of AES, which employs the MixColumns transformation involving a 4×4 matrix multiplication with a column vector over *GF*(2^8^). In this scenario the pre-computed lookup table solution presented by Gladman [22] proves to be the most efficient and optimal on a 32-bit register, requiring 4KB of memory. However, if we employ the proposed method to reduce storage to 2KB, the lookup table elements become 16-bit numbers. Consequently, operations are conducted on this register, leading to increased table accesses and XOR operations. Nonetheless, for larger lookup table sizes, like those in rows 5, 6, and 7 of Table 2, the effectiveness becomes evident as memory and table counts decrease, while the number of memory accesses and XOR operations remains constant.

## 7. Conclusion

In this article, we propose a new lookup table technique to reduce the storage memory when executing in the software. This technique applies to the linear layer of block ciphers with large MDS matrices such as recursive, Hadamard, or circulant ones. Accordingly, the proposed lookup table technique exploits the recursive properties of linear matrices, the similarity in the elements in each row of the Hadamard matrix or the circulant one, allowing the implementation of a linear layer with calculation complexity (number of XORs and memory accesses) to remain the same compared to the usual lookup table implementation. Furthermore, the proposed method allows a significant reduction in the memory for storing pre-calculated lookup tables, which can be reduced by up to 4 or 8 times or even more. The larger the size of the MDS matrices of the linear layer, the more benefits the proposed method has, which means the less storage memory it needs. Memory storage will decrease even more as the size of the MDS matrix increases. For example, when analyzing the case with a matrix size of 64, the memory storage ratio with the proposed lookup table technique decreases by 87.5% compared to the conventional lookup table technique. It is quite possible to apply the proposed approach to the case of a matrix for a complete block to increase the diffusion layer’s branch number or increase the security of the block cipher without raising the memory for the pre-calculated lookup tables. This method also allows more flexible implementation of large linear layers in different environments. Future research will focus on applying the proposed implementation method to practical ciphers using MDS matrices such as recursive, Hadamard, or circulant ones, and then we can accurately evaluate the efficiency of this method.

## Supporting information

S1 File (ZIP)
