# Supplementary material for: Efficient implementation of the linear layer of block ciphers with large MDS matrices based on a new lookup table technique
Source: PLoS One. 2024 Jun 21;19(6):e0304873. doi: 10.1371/journal.pone.0304873 (PMC11192358; doi:10.1371/journal.pone.0304873)
Supplement: S1 File — (ZIP) [file pone.0304873.s001.zip › Supporting Information files/Experimental implementation/Experimental Kuznyechik.docx]

**Implementation for the test case of Kuznyechik block cipher**

The implementation is in C++, compiled using Visual Studio 2022 on a computer with an Intel® Core™ i5-6200U CPU @ 2.30GHz 2.4 GHz, running Windows 10 64-bit version.

The method of implementing the lookup table is illustrated in the figure below (example for a $4\times4$ matrix case).

Figure 1. Example for a $4\times4$ matrix case

The parameter settings for the lookup table for the entire $16\times16$ matrix of the Russian Kuznyechik block cipher require the use of 16 lookup tables (*LS15, LS14, ..., LS0*), each table consisting of 512 elements of 64 bits (as shown in Figure 1 below). The total memory required is $16\times512\times8$ (bytes) = $65536$ bytes = $64$ Kbytes. If including both the encryption and decryption processes, then it would require $64\times2=128$ Kbytes. If considering 256 elements of 64 bits per table, each table needs to be divided into two tables. Thus, the number of tables at that point would be 32.


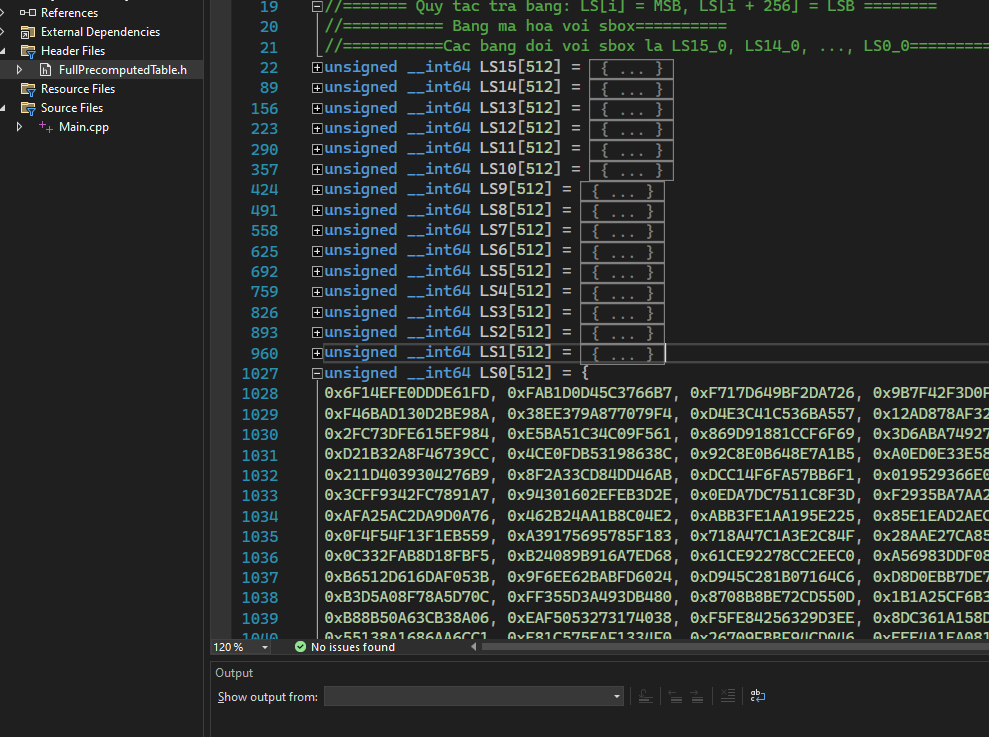


*Figure 2. Declaration of the lookup table for the encryption process of the Kuznyechik block cipher (configured for the full matrix)*

Figure 3 depicts the implementation using an approach to compute the lookup table for half the number of matrices according to our proposed method. We also require 16 tables *sLS15, sLS14, ..., sLS0*. However, each table only needs 256 elements of 64 bits. Thus, the memory requirement is halved compared to the full implementation as shown in Figure 2.


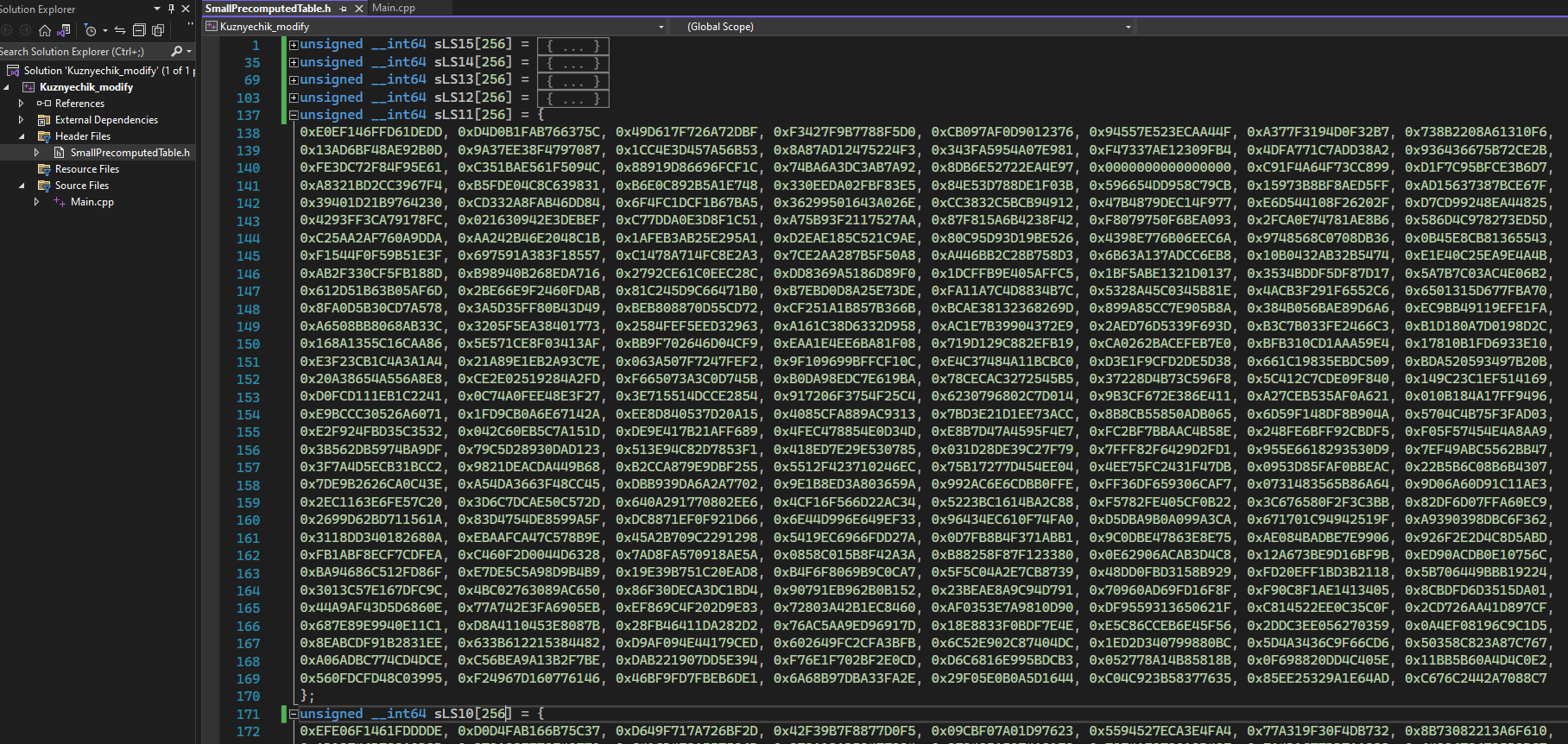


*Figure 3. Declaration of the lookup table for the encryption process of Kuznyechik (configured for half the number of columns in the matrix using our approach)*

Figure 4 and Figure 5 respectively represent the number of basic operations (number of XOR operations, number of table lookups) for the implementation using lookup tables for the full matrix and the implementation according to our proposed method.


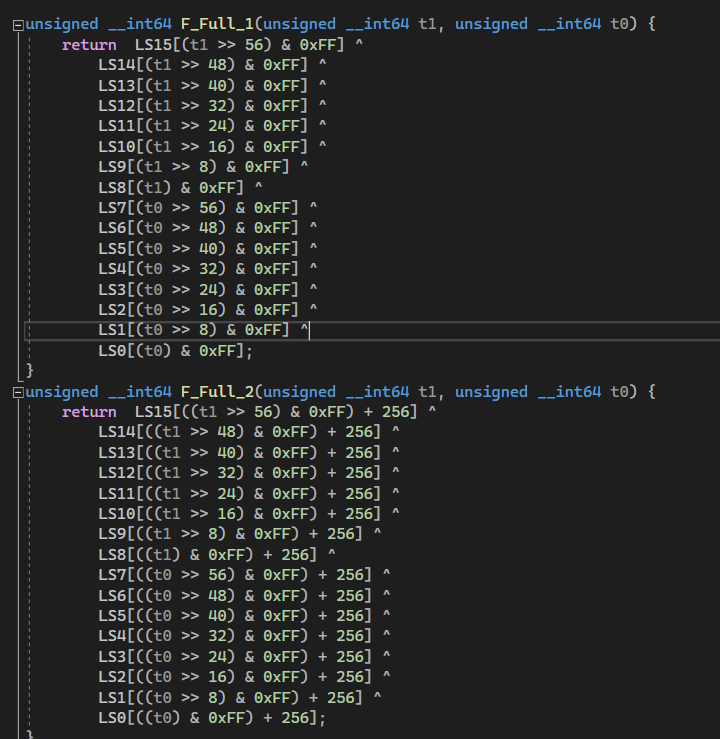


*Figure 4. The number of operations performed using the lookup table technique of Kuznechik (for the full matrix).*


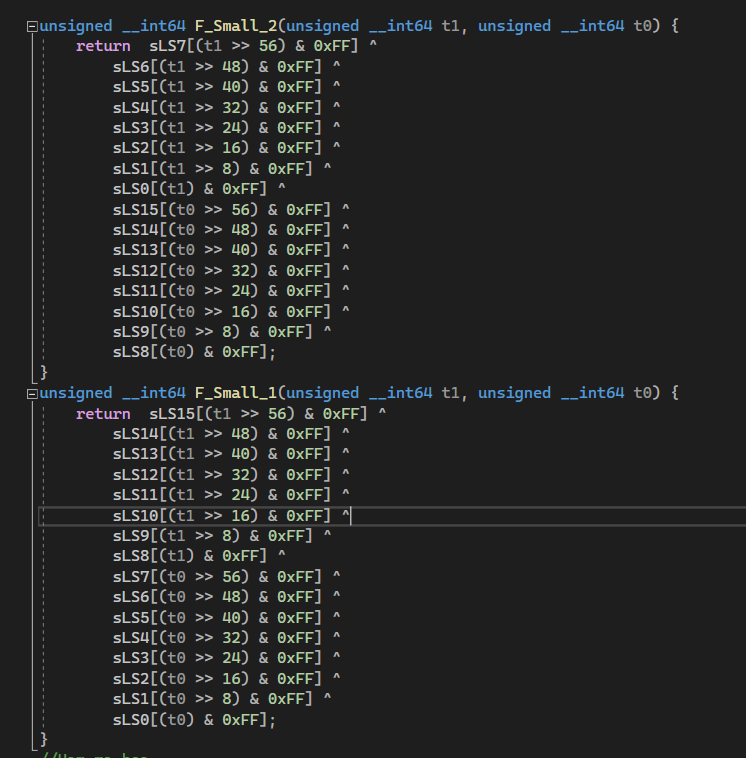


*Figure 5. The number of operations performed using the lookup table technique of Kuznechik (for half the number of columns in the matrix according to our proposal)*

We observe that the number of basic operations for both implementation methods is the same (as in the theoretical analysis).

Figure 6 shows the execution speed when applying the proposed implementation method and the implementation method using the full matrix for Kuznyechik: It is evident that the encryption speed of the proposed method is higher. The reason may be due to accessing a lookup table with fewer elements (256 elements for the proposed method and 512 elements for the implementation using lookup tables for the full matrix), resulting in faster processing speed. Although the number of accesses and the number of XOR operations are the same. The implementation is purely in C++, compiled using Visual Studio 2022 on a computer with an Intel® Core™ i5-6200U CPU @ 2.30GHz 2.4 GHz, running Windows 10 64-bit version.


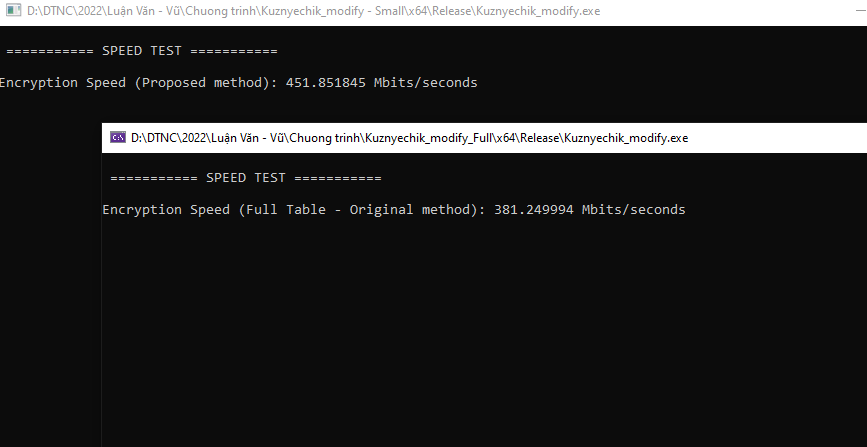


*Figure 6. Execution speed comparison between the proposed method and the implementation method using lookup tables for the full matrix applied to Kuznyechik.*
