# Supplementary material for: Efficient implementation of the linear layer of block ciphers with large MDS matrices based on a new lookup table technique
Source: PLoS One. 2024 Jun 21;19(6):e0304873. doi: 10.1371/journal.pone.0304873 (PMC11192358; doi:10.1371/journal.pone.0304873)
Supplement: S1 File — (ZIP) [file pone.0304873.s001.zip › Supporting Information files/Some MDS matrices are investigated/MDS matrices investigated.docx]

**Some MDS matrices are investigated**

**1. Recursive MDS matrix in the LED block cipher**


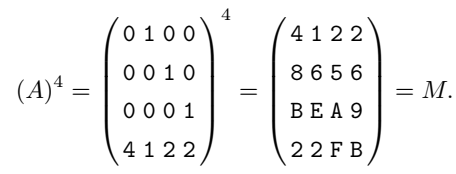


in which, the base field is $GF(2^{4})$ with the primitive irreducible polynomial $f\left( x \right)=x^{4}\oplus x\oplus1$.

**2. Recursive MDS matrix in the Kuznyechik block cipher**

It is also an MDS matrix with a size of $16\times16$ over the $GF(2^{8})$ field with the primitive polynomial $f\left( x \right)=x^{8}\oplus x^{7}\oplus x\oplus1$.


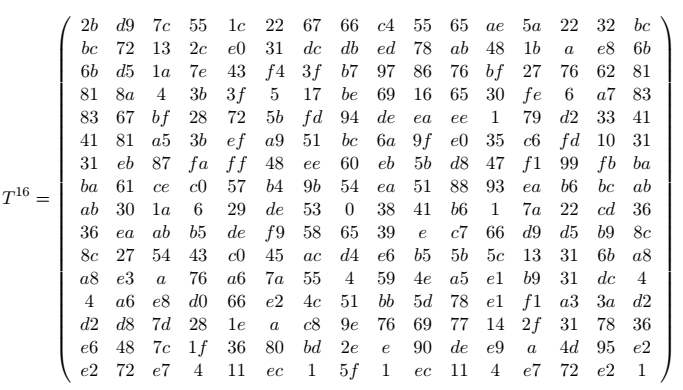


**3. Circulant MDS matrix in the Whirlpool hash function**


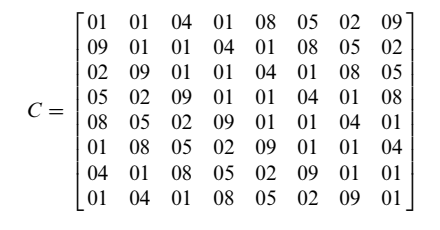


This is an $8\times8$ MDS matrix over $GF\left( 2^{8} \right)$, where the primitive polynomial is $f\left( x \right)=x^{8}\oplus x^{4}\oplus x^{3}\oplus x^{2}\oplus1$.

**4. Circulant MDS matrix of AES**


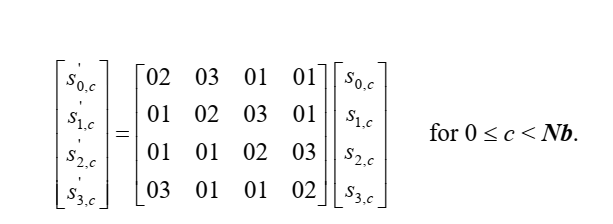


This is an $4\times4$ MDS matrix over $GF\left( 2^{8} \right)$ with $f\left( x \right)=x^{8}\oplus x^{4}\oplus x^{3}\oplus x\oplus1$.
