# Supplementary material for: Efficient implementation of the linear layer of block ciphers with large MDS matrices based on a new lookup table technique
Source: PLoS One. 2024 Jun 21;19(6):e0304873. doi: 10.1371/journal.pone.0304873 (PMC11192358; doi:10.1371/journal.pone.0304873)
Supplement: S1 File — (ZIP) [file pone.0304873.s001.zip › Supporting Information files/Resource Dependency Chart/Resource Dependency on Size.docx]

**Resource Dependency on Size**

**Table 1.** Comparison of general implementation parameters using the proposed lookup table method and conventional lookup table method.

| **Parameters** | **Method for implementation using the lookup table** | **Register size (bits)** | **Memory size requirement (in bits) for the lookup table** | **Number of lookup tables** | **Number of memory accesses** | **Number of XORs** | **Size of the linear transform** |
| --- | --- | --- | --- | --- | --- | --- | --- |
| $m= 2^{l},$  $l\geq4$,  $n= 8$ | For matrix $M$  (using the conventional lookup table) | *H* | $nm^{2}2^{n}$ | $\frac{nm^{2}}{h}$ | $\frac{nm^{2}}{h}$ | $\frac{nm^{2}}{h}$ | $mn$ |
|  | **For the matrix** $\boldsymbol{A}^{\frac{\boldsymbol{h}}{\boldsymbol{n}}}$**, or for** $\frac{\boldsymbol{h}}{\boldsymbol{n}}$ **the number of columns of** $\boldsymbol{M}$ **(when** $\boldsymbol{M}$ **is a Hadamard or Circulant matrix)**  **(using the proposed lookup table)** | ***H*** | $hm2^{n}$ | *m* | $\frac{nm^{2}}{h}$ | $\frac{nm^{2}}{h}$ |  |

**Table 2.** *Comparison of the parameters of the implementation using the proposed lookup tables and ones in [23, 32, 42]*

| **№** | **Parameters** | **Method for implementation using the lookup table** | **Register size (bits)** | **Lookup table size** | **Number of lookup tables** | **Number of memory accesses** | **Number of XORs** | **Size of the linear transform** |
| --- | --- | --- | --- | --- | --- | --- | --- | --- |
| 1 | $m= 4$  $n= 4$ | For matrix $M$  (For example, the matrix of LED [25]) | 16 | 128 B | 4 | 4 | 4 | 16 bits |
|  |  | **For matrix** $\boldsymbol{A}^{\boldsymbol{2}}$**, half the number of columns of** $\boldsymbol{H}$ **or** $\boldsymbol{C}$ | **8** | **64 B** | **4** | **8** | **8** |  |
| 2 | $m= 4$  $n= 8$ | For matrix $M$  (For example, the matrix of AES [22, 23]) | 32 | 4 KB | 4 | 4 | 4 | 32 bits |
|  |  | **For matrix** $\boldsymbol{A}^{\boldsymbol{2}}$**, half the number of columns of** $\boldsymbol{H}$ **or** $\boldsymbol{C}$ | **16** | **2 KB** | **4** | **8** | **8** |  |
| 3 | $m= 8$  $n= 4$ | For matrix $M$ | 32 | 4 KB | 8 | 8 | 8 | 32 bits |
|  |  | **For matrix** $\boldsymbol{A}^{\boldsymbol{4}}$**, half the number of columns of** $\boldsymbol{H}$ **or** $\boldsymbol{C}$ | **16** | **2 KB** | **8** | **16** | **16** |  |
| 4 | $m= 8$  $n= 8$ | For matrix $M$  (For example, the matrix of Whirlpool [46, 47], Streebog [42], Kalyna [43, 44]) | 64 | 16 KB | 8 | 8 | 8 | 64 bits |
|  |  | **For matrix** $\boldsymbol{A}^{\boldsymbol{4}}$**, half the number of columns of** $\boldsymbol{H}$ **or** $\boldsymbol{C}$ | **32** | **8 KB** | **8** | **16** | **16** |  |
| 5 | $m= 16$  $n= 8$ | For matrix $M$  (For example, the matrix of Kuznyechik [24]) | 64 | 64 KB | 32 | 32 | 32 | 128 bits |
|  |  | **For matrix** $\boldsymbol{A}^{\boldsymbol{8}}$**, half the number of columns of** $\boldsymbol{H}$ **or** $\boldsymbol{C}$ | **64** | ***32 KB*** | ***16*** | **32** | **32** |  |
| 6 | $m= 32$  $n= 8$ | For matrix $M$ | 64 | 256 KB | 128 | 128 | 128 | 256 bits |
|  |  | **For matrix** $\boldsymbol{A}^{\boldsymbol{8}}$**, a quarter of number of columns of** $\boldsymbol{H}$ **or** $\boldsymbol{C}$ | **64** | ***64 KB*** | ***32*** | **128** | **128** |  |
| 7 | $m= 64$  $n= 8$ | For matrix $M$ | 64 | 1024 KB | 512 | 512 | 512 | 512 bits |
|  |  | **For matrix** $\boldsymbol{A}^{\boldsymbol{8}}$**, one per eight of number of columns of** $\boldsymbol{H}$ **or** $\boldsymbol{C}$ | **64** | ***128 KB*** | ***64*** | **512** | **512** |  |

**Table 3.** *Lookup Table Size (KB) with corresponding parameters for* $n = 8,$ *m =*$2^{l}$

|  | $l$ | 4 | 5 | 6 | 7 |
| --- | --- | --- | --- | --- | --- |
|  | Full Matrix | 64 | 256 | 1024 | 4096 |
| $h = 32$ | one per eight of number of columns | 16 | 32 | 64 | 128 |
| $h = 64$ | Half the number of columns | 32 | 64 | 128 | 256 |

**Table 4.** *Number of lookup tables with corresponding parameters for* $n=8$

|  | $l$ | 4 | 5 | 6 |
| --- | --- | --- | --- | --- |
|  | Full Matrix | 32 | 128 | 512 |
| $h = 32$ | one per eight of number of columns | 16 | 32 | 64 |
| $h = 64$ | Half the number of columns | 16 | 32 | 64 |

**Fig 1. Correlation between matrix size and lookup table size (in Kbytes)**

**Fig 2. Number of lookup tables for various implementation types**
