# Supplementary figures and images for: Efficient implementation of the linear layer of block ciphers with large MDS matrices based on a new lookup table technique
Source: PLoS One. 2024 Jun 21;19(6):e0304873. doi: 10.1371/journal.pone.0304873 (PMC11192358; doi:10.1371/journal.pone.0304873)

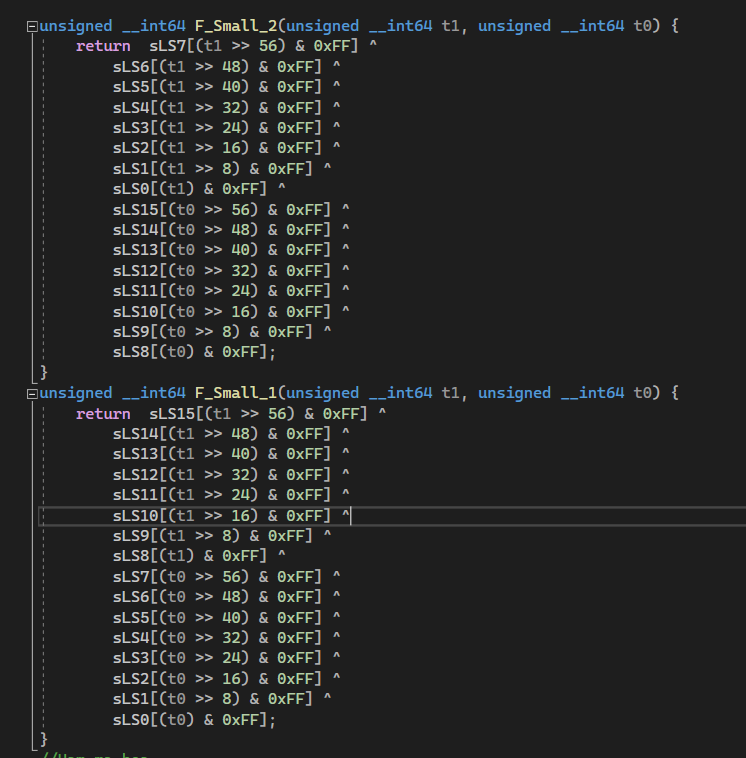

Supplement: S1 File — (ZIP) [file pone.0304873.s001.zip › Supporting Information files/Experimental implementation/4.PNG]

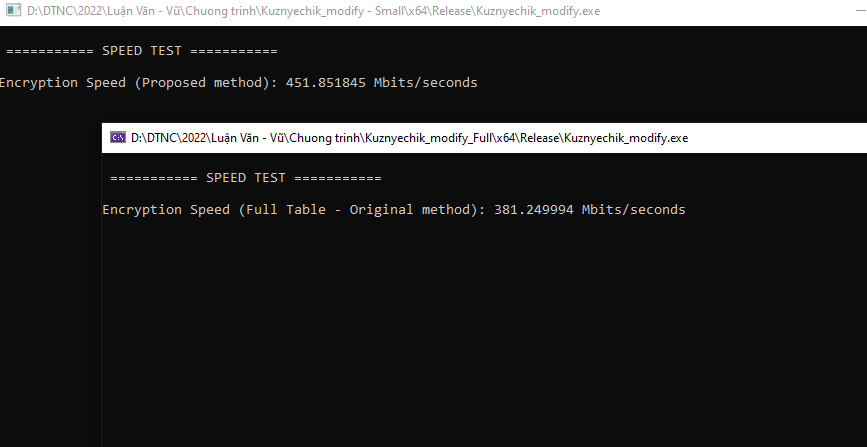

Supplement: S1 File — (ZIP) [file pone.0304873.s001.zip › Supporting Information files/Experimental implementation/5.PNG]

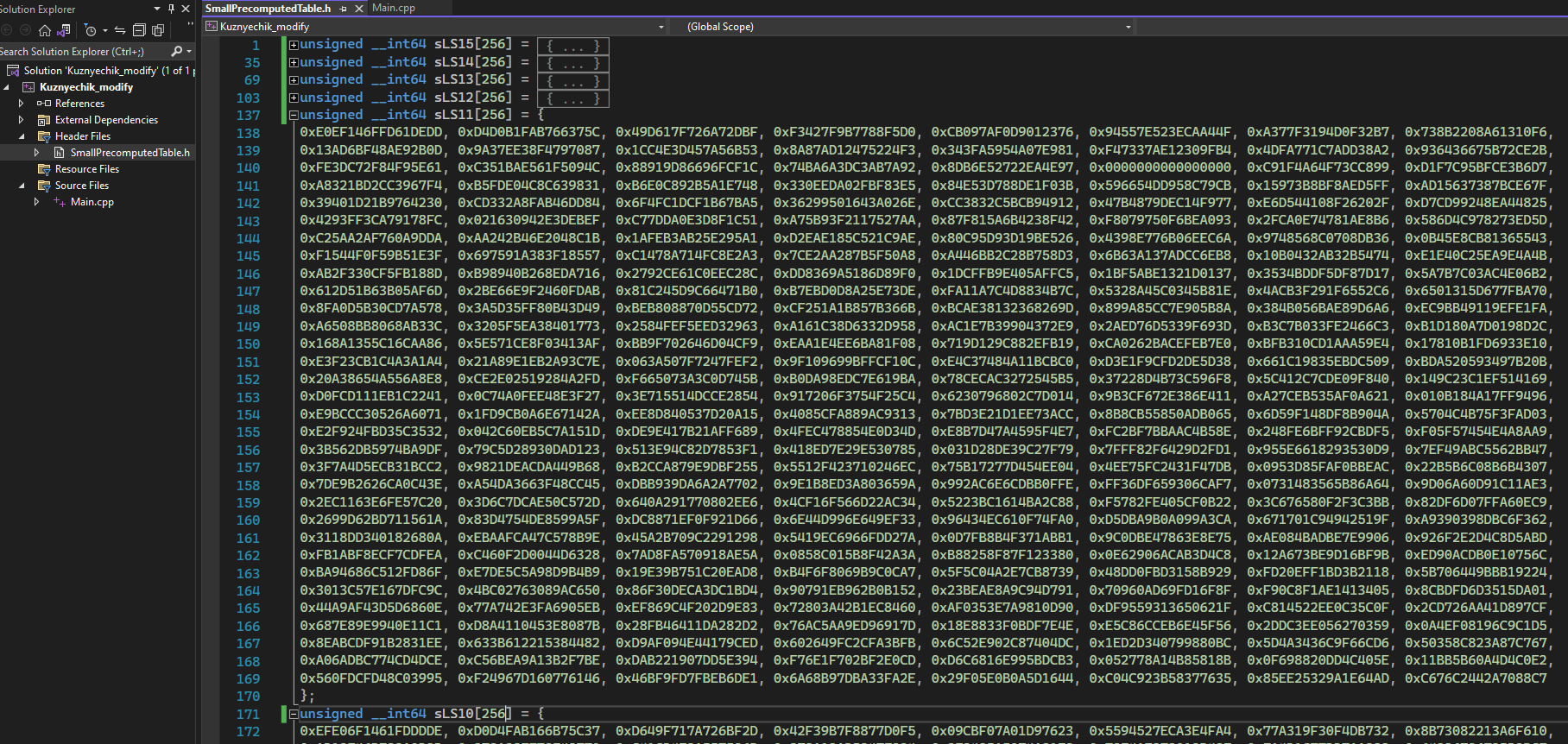

Supplement: S1 File — (ZIP) [file pone.0304873.s001.zip › Supporting Information files/Experimental implementation/2.PNG]

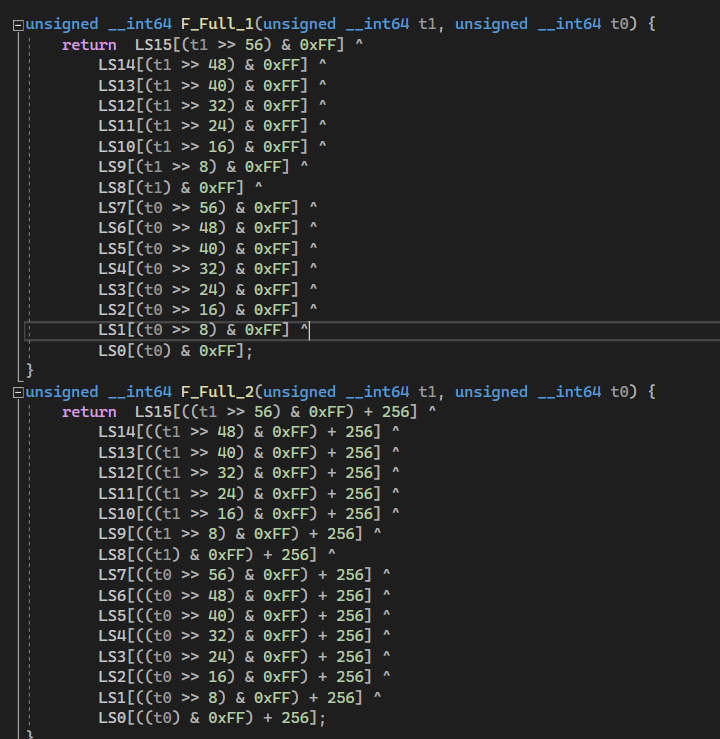

Supplement: S1 File — (ZIP) [file pone.0304873.s001.zip › Supporting Information files/Experimental implementation/3.PNG]

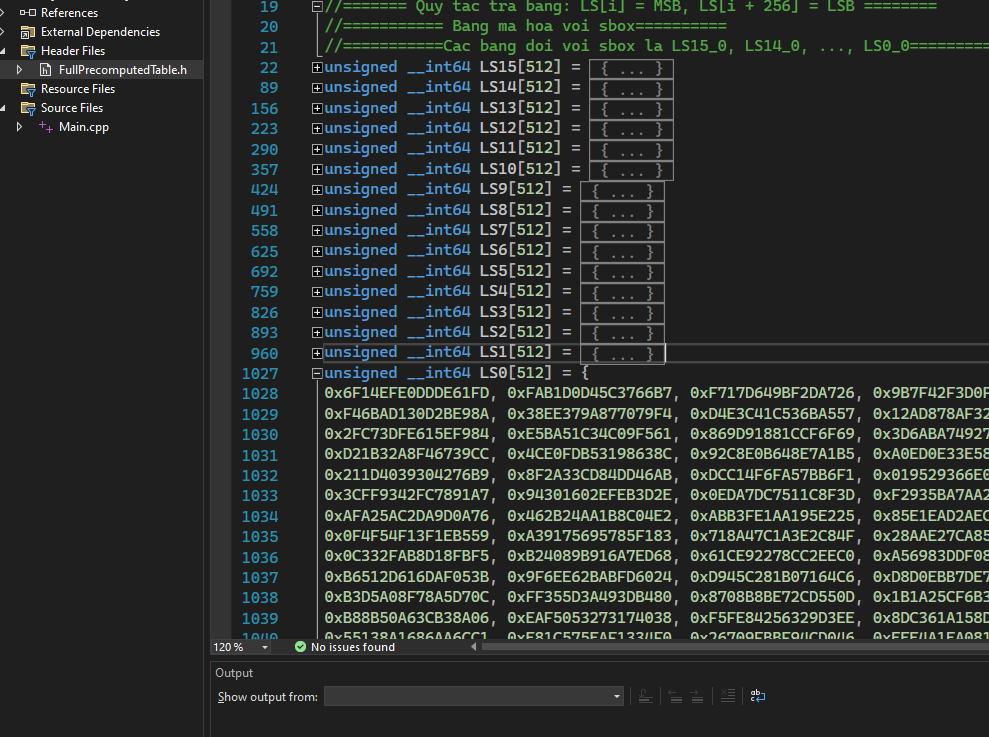

Supplement: S1 File — (ZIP) [file pone.0304873.s001.zip › Supporting Information files/Experimental implementation/1.PNG]
